# Aberrant right subclavian artery (arteria lusoria) aneurysm with a Kommerell’s diverticulum

**DOI:** 10.1590/1677-5449.009118

**Published:** 2019-02-28

**Authors:** Elif Coşkun, Levent Altınay, Anıl Tekin, Ufuk Tütün

**Affiliations:** 1 Bulent Ecevit University, Faculty of Medicine, Department of Cardiovascular Surgery, Zonguldak, Turkey.

**Keywords:** aberrant right subclavian artery, Kommerell’s diverticulum, vascular anomaly, artéria subclávia direita aberrante, divertículo de Kommerell, anomalia vascular

## Abstract

The treatment options for aberrant right subclavian artery vary depending on the presence of Kommerell’s diverticulum. Because there is a tendency not to report mortalities of these rare cases in the literature, it is hard to reach a conclusion on treatments from the limited data on post-interventional results in these patients. We report our experience with a 67-year old patient with an aberrant right subclavian aneurysm with Kommerell’s diverticulum, diagnosed by chance.

## Resumo

As opções de tratamento para artéria subclávia direita aberrante variam dependendo da presença de divertículo de Kommerell. Como há uma tendência a não relatar mortalidade nos raros casos descritos na literatura, é difícil chegar a uma conclusão sobre tratamentos tendo em vista os dados limitados sobre resultados pós-intervenção nesses pacientes. Relatamos aqui nossa experiência com um paciente de 67 anos de idade com aneurisma de artéria subclávia aberrante direita com divertículo de Kommerell diagnosticado ao acaso.

## INTRODUCTION

 Aberrant right subclavian artery (ARSA) is the most common congenital anomaly of the aortic arch. The cause of this anomaly is considered to be a disturbance of the right fourth pharyngeal arch which develops the innominate artery and persistence of the seventh intersegmental artery. 1 The incidence of left aortic arch anomaly with aberrant right subclavian artery is 0.7 to 2% in the population. 2

 Types of ARSA reported in the literature are as follows: retro-esophageal (80-84%), between trachea and esophagus (12.7-15%) and pre-tracheal (4.2-5%). 3 The ARSA is usually diagnosed by chance, as in our case. Barium radiography, esophagoscopy, and thorax computed tomography (CT) imaging techniques can be utilized for exact diagnosis of ARSA. 4 The location and course of the right subclavian artery, the degree of compression exerted on adjacent mediastinal organs, and concomitant vascular anomalies can be visualized in thorax CT angiogram. 4

 Open surgical repair and hybrid/endovascular intervention options are available for treatment of this pathology. The treatment decision should be taken based on the experience of the surgical team and the technical resources available at the health centre. Herein we represent a case of ARSA with Kommerell’s diverticulum in a patient who was lost because of intraoperative bleeding complications. 

## CASE DESCRIPTION

**Figure 1:**
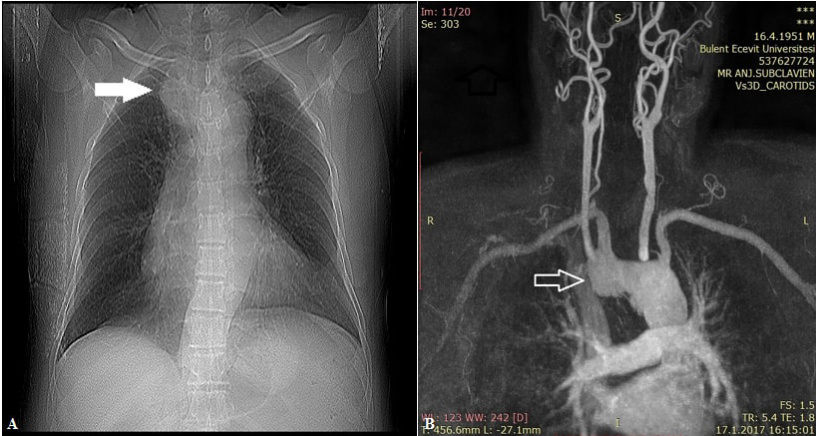
Chest radiogram and MRI of the patient.

**Figure 2:**
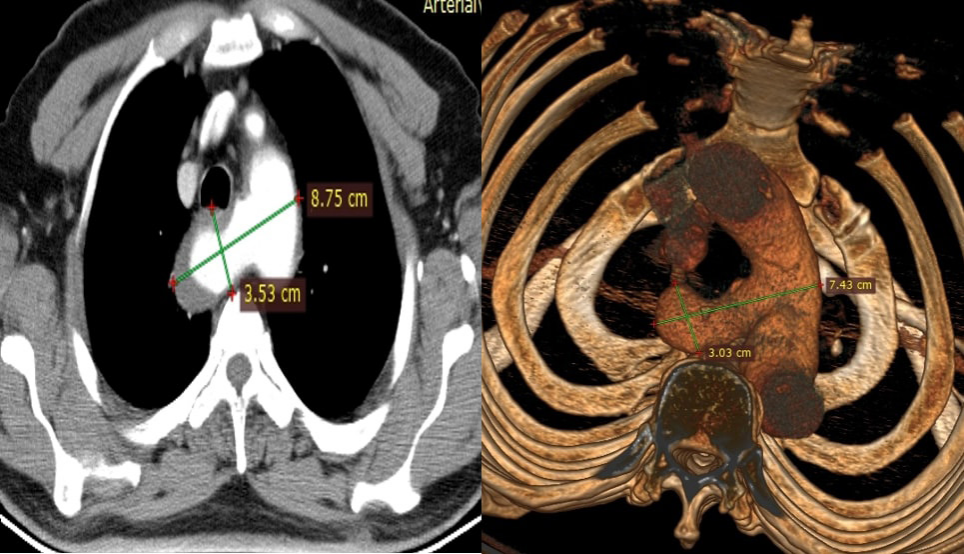
Thorax CT section and 3D image of the ARSA.

 A 67-year old male patient presented to the otolaryngology clinic with a swelling on the left side of his jaw which had been present for 12 years but had enlarged recently. His medical history included an operation for a swelling on the right side of his jaw at another centre, 17 years previously. However, there was no medical record of that operation. His recent medical treatment included doxazosin for hypertension and inhaler bronchodilator for chronic obstructive pulmonary disease. Pathology examination of the biopsy materials of the swelling excluded malignancy and the patient was scheduled for a parotidectomy operation with a diagnosis of benign Whartin tumor. He was a smoker for 55 years and an ex-coal mine worker and rhonchi were present in his physical examination. Therefore, chest radiography and magnetic resonance imaging (MRI) were performed. In these examinations, a 40 mm ARSA aneurysm was observed posterior of the trachea ( Figure 1 ). Thorax CT angiography with contrast was then performed to determine the relation of the aneurysm to adjacent organs ( Figure 2 ). 

 Although an endovascular intervention had been planned initially, because of the patient’s comorbidities, the anatomical measurements of the ARSA were inappropriate for placement of an endovascular stent so we decided to perform open surgery. We planned surgical resection of the Kommerell diverticulum through left thoracotomy and repair of the descending aorta with primary sutures or patching of the descending aorta with polytetrafluoroethylene (PTFE) graft. We were also going to implant the left subclavian artery into the left common carotid artery with fine running polypropylene sutures. 

### Operation technique

 Written informed consent was obtained from the patient and he was operated under general anesthesia. The chest cavity was accessed through the 4th intercostal space after left lateral thoracotomy. Sudden abundant bleeding from the posterior wall of the aneurysm occurred during surgical exploration of the aortic arch. The patient was lost because of the massive bleeding and hemodynamic instability. 

## DISCUSSION

 The diverticulum formed by the ARSA (lusorian artery) was first reported by a German radiologist, Dr. Burckhard Friedrich Kommerell, in 1936. 5 More recently, Backer et al. described it as an enlargement of the base of the subclavian artery over 1.5 times the diameter of the distal portion of the same artery in 2012. 6 Kommerell’s diverticulum can be seen in 20 to 60% of patients with abnormal right or left subclavian arteries. 3 There is still controversy about techniques for measurement of the diverticulum, because of the lack of specific data. Tsuki 7 and Idrees 8 suggested two different measurement techniques: (a) measurement of the cross-sectional diameter from the opposing aorta wall to the tip of the diverticulum; (b) measurement of the diameter of the abnormal subclavian artery at the site of origin in the aortic arch. The indication of surgery with regard to the size of the diverticulum is unclear because of the non-standardized measurement techniques. 

 The optimal surgical treatment advised for this pathology is carotid artery – subclavian artery bypass with a vascular graft following surgery to repair the artery with a synthetic graft under cardiopulmonary bypass. 9 Postoperative morbidity can include ischemic complications or subclavian steal syndrome after surgical reconstruction of the abnormal subclavian artery 10 and phrenic or recurrent nerve damage. 11 Major causes of mortality can be listed as follows: pulmonary embolism, bleeding, sepsis (mediastinitis), and chylothorax. 

 Endovascular treatment of ARSA is more common than traditional surgical procedures nowadays. 1 It does not require general anesthesia, can be done in a shorter time, is less invasive, and there is a lesser risk of blood loss due to intraoperative bleeding than with open surgery. However, there are some drawbacks of endovascular intervention such as endoleak, arterial-esophageal fistula and ipsilateral upper extremity claudication. 8 Besides these, the long term results of this treatment are still unknown. 9 Unfortunately, anatomic incompatibility is still a restricting factor for endovascular intervention. 

 Tassiopoulos et al. reported that the anatomic compatibility of ARSA for endovascular graft implantation is crucial for success. 12 Our case was incompatible with endovascular treatment because the length of the proximal aneurysm neck was insufficient. Therefore, we decided to do an open surgical repair in our case. 

 Several endovascular techniques are described in the literature: utilizing fenestrated aortic covered stents, 13 covering the ARSA with three barreled thoracic endovascular grafts, 14 and introducing a ‘periscope’ covered stent into the ARSA. 15 However, because of the high costs of branched/chimney grafts and anatomical unsuitability for standard endovascular interventions, we decided to perform open surgery in this case. 

 There is a tendency not to report mortalities of these rare cases in the literature. It is hard to reach an exact conclusion about the most feasible treatment method with the limited post-interventional data. We think that open surgical procedures are very challenging because of the anatomy of the pathology. Extreme care should be taken during exploration of the great vessels in the surgery. 

## CONCLUSION

 The exact anatomy of the pathology should be clearly determined. Endovascular treatment should be chosen in suitable cases; otherwise open surgery is the only option we have. 
